# Supporting safe walking and managing missing incidents in dementia: a qualitative narrative synthesis of current evidence

**DOI:** 10.1093/ageing/afaf371

**Published:** 2025-01-03

**Authors:** Hoi Tat Kwok, Annika Dhawan, Valerie Lye, Lawrence Fong, Phuong Leung, Vasiliki Orgeta

**Affiliations:** Division of Psychiatry, University College London, London, W1T 7NF, UK; Division of Psychiatry, University College London, London, W1T 7NF, UK; Division of Psychiatry, University College London, London, W1T 7NF, UK; Division of Psychiatry, University College London, London, W1T 7NF, UK; Division of Psychiatry, University College London, London, W1T 7NF, UK; Division of Psychiatry, University College London, London, W1T 7NF, UK

**Keywords:** dementia, safe walking, missing incidents, narrative synthesis, tracking devices, systematic review, older people

## Abstract

**Background:**

People with dementia are at risk of going missing while walking outdoors, which can significantly affect their safety and well-being. However, evidence on their lived experiences during such incidents, as well as on strategies that may improve care remain limited.

**Aim:**

To conduct a narrative synthesis of qualitative studies exploring the lived experiences and preferences of people with dementia, family carers and professionals in relation to strategies for supporting safe walking and preventing missing episodes in both community and residential care settings.

**Methods:**

Four electronic databases (Medline, Embase, PsycINFO and CINAHL) were systematically searched up to April 2025. Studies were appraised using the Critical Appraisal Skills Programme qualitative research checklist. A narrative synthesis framework was adapted to explore themes emerging within and among studies.

**Results:**

Of the 19 207 articles identified, 25 met the inclusion criteria. Four themes emerged: (a) emotional impact of missing episodes, (b) safe walking as a fundamental need for people with dementia, (c) existing strategies for safe walking and responding to missing episodes and (d) experiences of using technological solutions to prevent and manage missing incidents.

**Conclusions:**

Preventive and reactive strategies, including technological devices, can promote safe walking for people with dementia. There is an urgent need to embed safe walking plans within dementia care packages to mitigate harms associated with missing incidents. Initiatives that raise awareness of risk among people with dementia, carers and professionals should form a core component of clinical practice.

## Key Points

- Understanding the lived experiences of people with dementia, their carers and stakeholders is essential for preventing dementia-related missing incidents.
- Safe walking is a fundamental need for people with dementia and requires tailored care plans.
- Personalised safe walking plans are vital to both prevent and manage missing incidents in dementia care.
- There is an urgent need for interventions that assess the effectiveness of care plans to support safe walking.
- https://doi.org/10.1093/ageing/afaf289

## Introduction

Dementia affects more than 55 million people globally, with nearly 10 million new cases every year [1]. Outdoor and social activities play a crucial role in supporting the well-being of people with dementia [2]; however, such engagement may also elevate their risk of going missing [3]. In the context of dementia, a missing incident (or missing episode) is defined as a situation in which a person with dementia is absent from an expected location and their whereabouts are unknown to carers or professionals [4], thereby raising concerns about their safety and well-being.

Missing episodes are highly distressing for both people with dementia and their family carers [5]. Approximately 37% to 40% of people with dementia experience at least one episode of going missing [6, 7], which is associated with severe harm, including drowning, traffic accidents or even death [3, 8, 9]. Such incidents often result in premature admission to long-term care facilities for people with dementia, even when they could otherwise continue living independently in the community [10].

Due to these risks, physical restraints and medications are often used as protective and preventive measures in home care settings, frequently without the consent of the person with dementia, which can be detrimental to their well-being and health [11]. Promoting safe walking practices—strategies that support outdoor mobility while minimising the likelihood of missing episodes or harm—is critical for enhancing autonomy, and lowering societal costs associated with long-term care [10, 12].

Prior reviews have predominantly evaluated the effectiveness of strategies implemented in long-term care or laboratory environments, focusing mainly on the usability aspects of technological approaches [13, 14]. Nevertheless, a significant gap persists in capturing the lived experiences of people with dementia, their carers and other stakeholders—knowledge essential for designing effective, person-centred interventions to support safe walking and prevent missing episodes [15]. This understanding helps identify the most feasible management strategies, to protect people with dementia from harm [16].

Therefore, a key aim of this review was to synthesise existing evidence on the lived experiences and preferences of people with dementia, carers and professionals concerning strategies to support safe walking and prevent missing episodes, with the goal of generating comprehensive understanding that informs the development of person-centred and practically relevant interventions.

## Materials and methods

### Narrative synthesis

We employed a narrative synthesis approach to integrate findings from multiple qualitative studies [17, 18]. Narrative synthesis uses textual descriptions to summarise and interpret results, enabling exploration of intervention effectiveness—what works, why and how [15]. This approach allowed us to develop a practical framework grounded in the perspectives of people with dementia, carers and professionals, to inform actionable recommendations for safe walking and preventing missing episodes. We followed the Guidance on the Conduct of Narrative Synthesis in Systematic Reviews [18] and registered our review with PROSPERO (CRD42023474560).

### Search strategy and selection of studies

A systematic search was conducted across four electronic databases (Medline, Embase, PsycINFO and CINAHL) up to April 2025. Terms relevant to missing and dementia were used: (miss^*^ OR go^*^ missing OR lost OR wander^*^ OR track^*^ OR search resource^*^ OR safe return^*^ OR police) AND (dement^*^ OR Alzheimer^*^) (see Appendix S1). To ensure a comprehensive search, we conducted a supplementary manual hand search on Google Scholar using the relevant terms stated above to avoid missing relevant studies. Two authors (H.T. and A.D.) independently screened titles and full texts, with discrepancies discussed with a third author (V.O.).

### Inclusion criteria

The inclusion criteria were as follows: (1) qualitative studies focusing on the lived experiences of people with dementia, formal and informal carers, on safe outdoor mobility (safe walking) and preventing missing episodes and views of key stakeholders (dementia care professionals, law enforcement personnel), (2) published in English. Mixed-method studies that reported qualitative data were also included. Studies reporting on lived experiences of indoor walking (in the house, other building or care home), and those not published in English were excluded.

### Data extraction and quality assessment

Data were extracted using pre-defined tables, including author and location, research aim, recruitment source, sample, research methods and reported themes. H.T. (principal appraiser), A.D. and P.L. independently appraised studies using the Critical Appraisal Skill Programme (CASP) qualitative checklist [19], recommended by the Centre for Reviews and Dissemination [20]. A.D. and P.L. appraised 18 and 7 articles, respectively, each in parallel with H.T. Each article meeting the inclusion criteria received a score of 1 (meeting criteria) or 0 (criteria not met or insufficient information), classifying studies as poor (score ≤ 4), moderate (score 5–7) or high quality (score ≥ 8) based on their overall score [21]. For the tenth item (how valuable is the research described), a score of 1 was given when both reviewers agreed.

### Data synthesis

We applied the narrative synthesis framework [18] by systematically scoping and mapping existing theories and qualitative literature on managing dementia-related missing incidents [13]. Data were extracted from articles and tabulated for descriptive summaries and initial comparisons, covering author and location, research aims, recruitment sources, samples, data collection and analysis methods and reported study themes. Quality of studies was assessed using the CASP qualitative checklist [19], focusing on aims, methodology, robustness of data collection, ethical considerations and data analysis transparency.

## Results

### Search process

**Figure 1:**
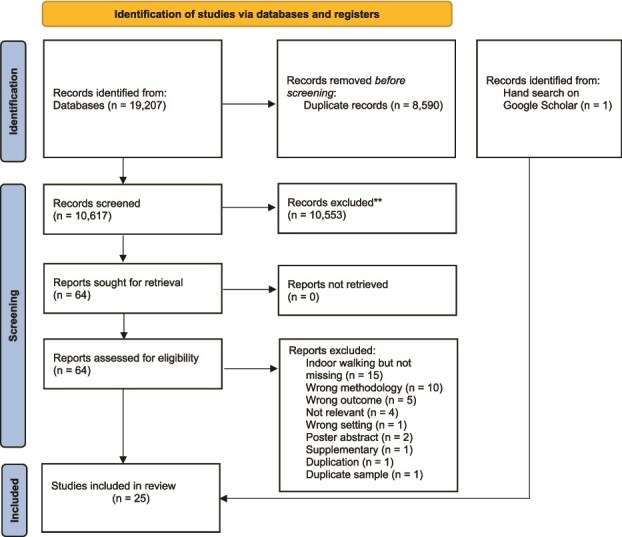
Flow diagram of the screening process.

A total of 19 207 articles were identified by the database search (see Figure 1 for Preferred Reporting Items for Systematic Reviews and Meta-Analyses flow diagram). After de-duplication, 10 617 full texts were retrieved for title and abstract screening, of which 10 553 studies were irrelevant, leaving 64 articles assessed for full eligibility. Of these, 40 studies were excluded with reasons (see Appendix S2), such as reporting on indoor walking but not missing incidents (*n* = 15) and wrong methodology (*n* = 10). A total of 25 studies met the inclusion criteria, of which one article was identified through a hand search on Google Scholar.

### Synthesis of findings

#### Study characteristics

The included 25 studies were conducted in the United Kingdom (*n* = 7), Canada (*n* = 7), United States (*n* = 3), Taiwan (*n* = 2), Korea (*n* = 1), Belgium (*n* = 1), Germany (*n* = 1), Israel (*n* = 1), Netherlands (*n* = 1) and Sweden (*n* = 1). Most were primarily qualitative (*n* = 19), with six using mixed methods [22–27]. Data collection included individual semi-structured interviews (*n* = 14), focus groups (*n* = 7), both methods (*n* = 3) and one observational study. Most studies explored perceptions (*n* = 6) and lived experiences (*n* = 5) of technology use to prevent missing episodes, walking risk mitigation (*n* = 2), technology design and ethics (*n* = 4), lived experiences of missing episodes (*n* = 2), safe walking perceptions and behaviour (*n* = 3), reporting of missing incidents (*n* = 1) and search and rescue strategies (*n* = 1).

#### Participant characteristics

A total of 688 participants were included across 25 studies, comprising 77 people with dementia, 237 family carers and 179 health and social care professionals. Some studies recruited older people without cognitive impairment (*n* = 7), police and law enforcement professionals (*n* = 64), community providers (*n* = 65), engineers (*n* = 6) and technological manufacturing representatives (*n* = 40). One study recruited researchers (*n* = 11) and government personnel (*n* = 2). Notably, one study included older people without cognitive impairment living in a care home [28], while people with dementia and family carers lived in community settings.

Sample sizes ranged from 5 to 109, with 11 studies involving people with dementia. Recruitment primarily occurred via healthcare services or voluntary sector organisations, with developers of electronic tracking devices recruited from corporate companies. Table 1 provides detailed study characteristics.

**Table 1:** Characteristics and summary of findings of included studies

Authors and location	Research aims	Recruitment source	Sample	Method of data collection and data analysis	Reported themes as stated in the study
Shalev-Greene *et al.* [34] UK	Identify factors influencing family carers’ reporting of missing incidents to the police	Non-governmental organisations	20 family carers (F = 7; M = 5; spouses = 7; parent–child = 4; child-in-law = 1) caring for people with dementia living in the community	1. Semi-structured interviews 2. Thematic Analysis	1. Understanding carers’ definition of missing incidents 2. Reporting missing incidents 3. Decision-making involved in reporting a missing incident to the police 4. Factors prompting carers to call the police: 4a. Protecting the person from risk 4b. Expecting the police to be helpful and successful 5. Factors inhibiting contacting the police: 5a. Embarrassment and guilt 5b. Fear of disapproval or judgement by the police 5c. Fear of negative reactions by the person with dementia 5d. Distrust of the police and desire to protect their relative
Kearns *et al.* [28] USA	1. Explore the effectiveness of technologies for managing missing incidents 2. Identify key elements of technologies	University hospital	43 participants (nursing home residents = 7; family carers of people with dementia = 7; home healthcare staff *n* = 9; long-term care staff = 7; medical staff = 7; engineers working in rehabilitation settings = 6)	1. Focus groups 2. Logic of discovery to develop concepts, generalisations and theories	1. Indoor technologies identified: motion detectors and weight-sensitive mats 2. Outdoor: Global positioning system-based devices for managing walking behaviour
Landau *et al.* [45] Israel	Ethical aspects of using the Global Positioning Systems (GPS)	Psychogeriatric clinics and the voluntary sector	68 carers (professional carers = 32; family carers = 36) caring for people with a diagnosis of dementia living in the community	1. Focus groups 2. Deductive and Inductive Thematic Analysis	1. Professionals’ views of the use of tracking devices: 1a. The need to ensure patient safety and autonomy 1b. Design should facilitate patient use 1c. Openness to new technology 2. Family carers’ views of tracking devices: 2a. Feasibility of using GPS depends on the stage of dementia 2b. Concerns about explaining their use to patients 2c. Protection of life is more important than privacy 2d. Favour toward body implanting electronic chip for tracking purposes
Neubauer *et al.* [16] Canada	1. Determine strategies used by stakeholders to prevent missing episodes 2. Describe risk mitigation strategies 3. Identify factors that influence the adoption of risk mitigation strategies	Identified by Alzheimer Societies	38 participants (people with mild dementia = 6; family carers = 10; paid carers = 7; social workers = 4; law enforcement officers and search and rescue personnel = 5; community staff = 4; occupational therapist = 1; geriatric psychiatrist = 1)	1. Semi-structured interviews 2. Direct content analysis and development of initial coding scheme based on interview guide	1. Risk mitigation strategies used for managing missing incidents: Commercial and phone locating devices (GPS) Home alarm systems, locks and barriers Phone maps and wayfinding Alerts to transit alarms distraction/redirection Surveillance and Identification tools Registry Supervision and calming techniques 2. Factors that influence adoption of risk mitigation strategies Education of risk perception and other resources Misconceptions, awareness and supervision Environmental elements Experience of people with dementia, their carers and the clinician Balance between risk and safety Perceived risk influence strategy adoption
Neubauer and Liu. [24] Canada	Developed and validated a conceptual model and series of guidelines for missing incidents	Voluntary sector and professional networks	38 participants (people with dementia = 5; formal carers = 2; long-term care nurses = 4; family carers = 4; staff at community organisations = 13; first responders = 4; clinicians = 2; social workers = 4)	1. Semi-structured interviews and focus groups 2. Directed content analysis	1. Perception of the conceptual model and guidelines 1a. Proactive guidance to help users decide what strategies keep people with dementia safe 1b. Reader friendly guideline to understand the risk level of people with dementia going missing 2. Changes that need to be made 2a. Terminology of guidelines 2b. Additional factors that influence strategies for wayfinding 2c. Additional visuals on guideline to be reader-friendly
Bantry-White *et al.* [29] UK^a^	1. Explore carers’ experiences of using electronic tracking 2. Elicit the values, beliefs and contextual factors that motivate use of electronic tracking 3. Explore the involvement of the person with dementia in decision-making and identify ethical issues 4. Describe the use of GPS tracking for people with dementia and generate hypotheses about impact	From a GPS tracking service provider; participants had either bought the device or had a free trial offered by the provider	10 family carers (partner = 6; adult-child = 4) caring for people with dementia living in the community; 9 out of 10 carers had reported the person missing in the last 6 months	1. Individual interviews and questionnaire to collect background characteristics 2. Thematic Analysis	1. Values informing decision-making 1a. The primacy of safety, independence and freedom 2. Context of decision-making 2a. Personality and relationship factors 3. Decision-making 3a. The role of the person in decision-making 3b. Process of decision-making in using electronic tracking 3c. The case of covert usage 4. Decision to use tracking 4a. Assessing safety 4b. Safety as paramount 4c. Personality and relationship context 4d. Accessibility and acceptability 5. Usage: Tracking as a primary and secondary intervention 6. Perceived impact 6a. Reassurance, independence and freedom 6b. Re-evaluation of safety concerns 6c. Reliability and limitations of tracking devices
Robinson *et al.* [25] UK	Explore the perspectives of key stakeholders in management of walking behaviour in dementia	From voluntary agencies and health care providers	19 participants (health and social care professionals = 10; family carers = 3; people with mild dementia = 6)	1. Focus groups 2. Thematic framework approach using deductive and inductive approaches	1. Conflicting perspectives on risk of missing incidents 1a. Carer perspective: Balancing rights and risks 1b. People with dementia perspective: Enjoying the fresh air 2. Conflicting perspectives on the process of risk assessment and management of missing incidents 2a. Use of electronic tracking devices: Conflict between autonomy and liberty vs. safety and security
Robinson *et al.* [30] UK	Create acceptable and effective technologies to facilitate independence for people with dementia	From voluntary sector organisations	25 participants (people with mild to moderate dementia = 10; family carers = 11; Alzheimer’s Society volunteers = 4	1. Focus groups 2. Constant comparative analysis	1. Perspectives of people with dementia: 1a. Independence 1b. Getting lost 2. Carers’ perspectives 2a. Change of routine 3. Participants’ views on existing technologies 3a. Challenge of incorporating technology into everyday life 3b. Weight and size of devices 3c. Feeling ‘tagged or tracked’
Dickson [31] USA	Examined family carers’ perceptions of people with dementia’s walking behaviour	From voluntary sector organisations	22 family carers caring for people with Alzheimer’s disease and related dementias	1. Semi-structured interviews 2. Grounded theory approach	1. Factors that impact carer’s perceptions 1a. Home safety and risk of falling 1b. Walking as a safety concern 1c. Burden on caregiving
Mahoney and Mahoney [43] USA	Research the state-of-the-art in wearable technologies for tracking and identify challenges for people with dementia and carers	From corporate companies	7 leaders from corporate companies	1. Semi-structured interviews 2. Unspecified analysis method	1. The wearability of the product 2. People with dementia’s perception of tracking devices 3. Adaptability of tracking devices for people with dementia 4. Features of tracking devices
Milne *et al.* [23] UK	Explore the perceived utility and challenges of GPS location	From social-care services	35 participants (people with dementia = 8; family carers = 12; social service staff = 5; occupational therapists = 4; community psychiatric nurse = 1; call centre operators = 2; police officers = 3)	1. Semi-structured interviews and focus groups 2. Thematic analysis	1. Perceptions of the concept of missing incidents and safer walking 2. Perceived utility and acceptability of using GPS technology
Howes *et al.* [42] Belgium	Gain an understanding of Electronic Tracking Devices (ETD) developers’ perceptions on ethical issues surrounding the design, development and use of these devices within dementia care	Sending emails to commercial companies and university consortiums and academic researchers working on ETD prototype development	15 developers of electronic tracking devices (F = 2; M = 13); age (29–71)	1. Semi-structured interviews 2. Constructivist grounded theory	1. Motivation to pursue of ETD development and justification of its use 1a. Motivation to intervene for the good 1b. ETDs are justified and unjustified in certain context 2. Technical and moral goals 2a. Evaluation of current ETD offering in dementia care 2b. Technical goal: Simplicity and Personalisation 2c. Moral goal for people with dementia and their carers 3. Conflicting values in decision making 3a. Conflicting ethical values, doing good and avoiding harm 4. Achieving balance 4a. Stakeholder inclusion in development 4b. Sources of moral input in developing ETD 5. Perceptions of moral responsibility 5a. Strong and week feelings of responsibility 5b. Boundaries of responsibility and misuse of ETDs
Dodds. [37] UK	Establish the types of walking behaviour carers experienced, how they dealt with this behaviour and ways of coping	From day centres	6 family carers (Spouse = 3; Daughters = 2; sister = 1) caring for people with a diagnosis of dementia	1. Semi-structured interviews 2. Unspecified analysis method	Coping Strategies: 1. Physical and chemical restraints to stop people walking out of the home 2. Diversion through activity 3. Avoidance or segregation from the relative 4. Collusion with wrongly held beliefs 5. Reality orientation 6. Verbal and physical aggression toward the person 7. Ignoring the behaviour 8. Involvement of others
Rasquin *et al.* [33] Netherlands	1. Provide an overview of the different strategies for preventing missing incidents 2. Explore technical support required for the use of electronic tracking devices	From health care institutions	25 participants (family carers = 15; professional carers =10) caring for people with dementia	1. Interviews and field pilot experiment 2. Unspecified analysis method	1. Missing leads to dangerous situations 2. Solutions to prevent missing episodes in people with dementia 3. Feasibility of using mobile phones with GPS and other technical devices 4. Issues encountering while using the technology
Neubauer *et al.* [41] Canada	1. Identify factors related to acceptance and usability of locator devices that are important to people with dementia, their care partners, service providers and technology developers	Professional networks	21 participants (service providers = 6; people with dementia = 5; technology developers = 5; family carers = 5)	1. Focus Groups 2. Qualitative description and conventional content analysis	Aspects in the acceptance and usability of locator devices 1. Inclusivity 2. Simplicity 3. Features 4. Physical properties 5. Ethics
Neubauer *et al.* [44] Canada	Describe the outcomes of a Locating Technology and Dementia Forum	From voluntary sector	109 participants (people with dementia = 3; care partners = 11; Alzheimer Society representatives = 32; community organisations serving older adults = 12; police officers and search and rescue teams = 19; healthcare services providers = 6; technology industry = 13; researchers = 11; government representatives = 2)	1. Focus groups 2. Qualitative content analysis	1. Prevention strategies: Proactive rather than reactive approaches to prevent risk 2. More knowledge (research) on risk of going missing and how it varies across disease stages 3. Learning more about specific locating devices/technologies 4. More conversations/forums about use of technology 5. Need to ensure technology is affordable or cost is supported
Liu *et al.* [22] Canada	Examine the acceptance of Global Positioning System (GPS) to prevent missing incidents	From health care services	24 participants (family carers = 15; stakeholders including home care case managers; occupational therapists; social workers; representatives from Alzheimer Society; Police officers = 9)	1. Focus groups 2. Unspecified analysis method	1. The potential usefulness of the GPS in locating the person 2. Experience in use of GPS for tracking and communication 3. Opportunities for improving devices
Olsson *et al.* [32] Sweden	Describe strategies such as outdoor walks to prevent missing episodes	From memory clinics and carers support centres	5 people with dementia that had a previous missing episode (F = 2; M = 3; age 55–73)	1. Ethnographic-inspired approach repeated observational study 2. Qualitative content analysis	Walking strategies 1. Landmarks 2. Using senses 3. Stopping, looking around and thinking 4. Walking the same way or loop in familiar areas 5. Only walk in places and on routes where they could see other people and houses
Adekoya *et al.* [38] Canada	Identify and discuss the implication of public disclosure of person information in an alert system for people living with dementia at risk of going missing	From research team’s existing professional network (Alzheimer societies, dementia advocacy organisations and first responders)	19 participants (people with dementia = 3; family carers = 5; search and rescue and police officers = 4; service providers = 7; F = 10; M = 9)	1. Semi-structured interviews 2. Conventional content analysis	1. Right to autonomy 2. Safety versus privacy 3. Stigmatisation 4. Informed and knowledgeable consent
Ah [36] Korea	Explore the experiences and efforts of police officers in charge of missing cases	From National Police Agency	16 police officers who involved in missing person cases (F = 1; M = 15)	1. Semi-structured interviews 2. Content and thematic analysis	1. The police search procedure for missing individuals with dementia 2. Current search strategies 3. Challenges in the search process 4. Further initiatives to prevent going missing again
Doyle *et al.* [26] UK	Evaluate whether wearers and carers found the tracker usable and practical, while acceptable in terms of independence for the wearer and reduced burden of the carer	From memory service and police force	31 participants (people with dementia = 14; family carers = 14; stakeholders from police and memory services = 3)	1. Qualitative interviews and focus groups 2. General inductive approach	1. Experience of carers 1a. Effective tracking and communication, finding the person quickly 1b. Social connection 1c. Reduced stress and anxiety and police involvement 2. Experience of people with dementia 2a. Improved independence 2b. Confidence to do usual activities and maintain social connection 2c. Managing anxiety and preventing the risk of harm 3. Impact of GPS features 3a. Loudspeaker 3b. Tracking app, geofencing and live updates 3c. Fall detection, low battery warning and save history
Hu *et al.* [39] Taiwan	Explore healthcare providers (HCPs) difficulties and strategies when caring for community-dwelling people with dementia who are at risk of going missing	From community healthcare organisations (home care agencies, outpatient departments, day care services)	25 healthcare providers (nurses = 11; occupational therapists = 2; paid carers = 5; physician = 2; physical therapists = 1; clinical psychologist = 1; dementia case managers = 3)	1. Semi-structured interviews 2. Thematic analysis	1. Difficulties faced by HCPs 1a. Disturbance caused by behaviour and psychological symptoms of dementia (BPSD) 1b. Difficulty in helping older family carers to keep the person from going out 1c. Difficulty in changing the attitudes of family members 1d. Families’ unawareness of the risk of getting lost strategies used by HCPs 2. Strategies used by HCPs 2a. Detecting the risk of getting lost through early assessment 2b. Encouraging the family to use resources or devices to prevent going missing 2c. Educating the family to manage BPSD 2d. Strengthening the patient’s crisis awareness
Letts *et al.* [27] Canada	Understand the current practices for return discussions with people living with dementia	From professional networks and associations and social media	20 participants (police officers = 9; search and rescue managers = 2; social workers = 2; programme managers = 4; other service provider = 3) working with people with dementia	1. Semi-structured interviews 2. Conventional content analysis	1. The purpose of the practices 1a. Check on well-being and safety 1b. Collect information for future use 1c. Offer support 2. The features of the practices 2a. Who is involved in return discussions, when and where they take place 2b. What is explored during a return discussion 3. Facilitators for return discussions 3a. System and organisation level 3b. Person and family level
Li *et al.* [35] Taiwan	Explored the experiences of family carers caring for people with dementia who were lost outside their homes	From outpatient departments of a regional teaching hospital and a community day care centre	20 family carers who took care of people with dementia (F = 15; M = 5; age 22–87)	1. Semi-structured interviews 2. Descriptive phenomenological approach	1. Surprised people with dementia lost outside 1a. People with dementia disappear unexpectedly 1b. Nervously looking 1c. Worried about persons with dementia getting lost or having accidents 2. Using strategies to prevent people with dementia from getting lost 2a. Someone supervises the person at all times 2b. Set up confinement to prevent the person from going out 2c. Contact neighbours and friends to prevent the person from getting lost 3. Using strategies to find people with dementia 3a. The person carrying contact information and use an electronic tracking device 3b. Find the person in familiar places 3c. Call the police to find the person 4. Exhaustion in long-term care for people with dementia 4a. Long-term stress and insufficient support from families 4b. Conflict between work and care responsibilities 4c. Reluctance to place people with dementia to long-term care institutions 5. Coping with the care load 5a. Learn strategies to prevent people with dementia from getting lost 5b. Family-assisted care work 5c. Using care replacement workers
Löbe and Petersen [40] Germany	Explores how people with dementia and their carers assess the potential, opportunities and risks of intelligent assistive technologies	From local hospitals and nursing homes	27 participants (people with dementia = 12; family carers = 15)	1. Semi-structured interviews 2. Structural qualitative content analysis	1. Using GPS bracelet to locate the person 1a. Independence 1b. Safety 1c. Privacy

^a^This study uses the same sample as Bantry-White et al. (2010). The earlier publication was excluded due to duplication.

#### Robustness of the synthesis

Quality was rated as moderate for 4 studies and high for 21 studies. Most studies clearly stated their aims, recruitment, data collection and main findings and were judged as valuable research. However, only three examined researcher–participant relationships, and nine lacked ethical approval details. See Appendix S3 for quality ratings.

### Themes

Narrative synthesis identified four overarching themes: (1) emotional impact of missing episodes, (2) safe walking as a fundamental need for people with dementia, (3) existing strategies for safe walking and responding to missing incidents and (4) experiences of using technological solutions to prevent and manage missing incidents.

### Theme 1: Emotional impact of missing episodes

Missing incidents were described as causing significant emotional distress for both people with dementia and their carers [29]. Participants described situations where the person would no longer walk independently due to a missing episode. Individuals recalled episodes where getting lost led to lasting fear for families. Limiting outdoor walking due to a missing incident was often perceived as contributing to social isolation:

‘*For six years I never left the house…I was scared* [24].’

Some people with dementia, however, lacked awareness of the risk of going missing and continued their pre-morbid walking habits despite experiencing a missing episode:

‘*I used to walk for miles, but my family don’t want me to go away far…they’re frightened, but I’m not* [30].’

Carers, on the other hand, expressed ongoing anxiety about missing episodes, particularly in cases where the person had gone missing more than once. They described how difficult it was to walk together with their relatives due to other ongoing caregiving responsibilities:

‘*You do hear such horrific stories of old people going missing and never being found again* [23].’

‘*He did get lost a couple of times…go on a walk by himself…I was really busy* [31].’

### Theme 2: Safe walking as a fundamental need for people with dementia

Across four articles, safe walking emerged as a central concept, with people with dementia describing it as enjoyable, integral to daily life and beneficial for physical health, stress reduction and promotion of independence:

‘*I want to feel as if I’ve got a bit of independence. . .I can I just go out* [25].’

Most people with dementia expressed confidence in their ability to navigate their environment and considered walking as part of their daily routine:

‘*Sometimes I stop for a cup of tea…then come back* [30].’

However, safety was a recurring concern. Carers described creating ‘safe areas’ through geofencing enabled by GPS devices, supporting autonomy while safeguarding the person with dementia. An advantage was that carers received alerts when their relative ‘exited’ or moved far from a safe area, allowing timely responses [23, 26].

### Theme 3: Existing strategies for safe walking and responding to missing incidents

People with dementia discussed how they often adopt specific strategies to prevent missing episodes, such as using familiar landmarks, walking only in well-known areas and relying on environmental cues while outdoor walking. Weather conditions and limited daylight were common barriers to their wayfinding abilities:

‘*The sun helps you find your way...if it’s cloudy…you don’t recognise things the same way when it’s dark* [32].’

However, relocation to unfamiliar environments could disrupt these strategies, prompting families to consider electronic tracking devices as a safety measure to monitor the person’s location in real-time [33]. When missing episodes occurred, carers typically viewed reporting these to the police as a last resort [34, 35], often done only after the person had been missing for an extended period:

‘*I didn’t know what to do…so ringing them (police) for advice…she’s been gone for two and a half hours*… [34]’

Police officers adopted various investigation and search techniques—including search dogs, helicopters, drones and CCTV footage—based on standardised guidelines to locate missing people [36]. Although safeguarding concerns often triggered reporting, many carers hesitated to report incidents to the police due to feelings of shame or guilt, or apprehension about potential impacts on the caregiving relationship; this was often related to people with dementia experiencing embarrassment at being reported:

‘*if she was upstairs in a neighbour’s…suddenly she comes downstairs…half the police force there…my mum was very embarrassed about that* [34].’

Police officers highlighted that carers’ previous interactions with police could influence their help-seeking behaviours:

‘*There’s also the fear of the police…if they’ve emigrated from a different country where the police were maybe not as trusted…* [24].’

Other than the police, carers tended to seek support from neighbours when the person with dementia experienced a missing episode [35, 37]. Carers commented that a community approach works better in rural areas where everyone knows each other:

‘*everyone knew who she was…in a small town and people would sort of help each other…* [24].’

Paid carers, however, noted that seeking help from neighbours in urban areas was difficult due to weak community connections [24]. Some studies described community alert systems designed to notify professionals and locals when someone went missing, facilitating a coordinated search [38]. Healthcare professionals emphasised follow-up discussions as a preventative measure, as these helped reduce recurrent incidents by identifying ways to prevent future missing episodes [27, 39].

### Theme 4: Experiences of using technological solutions to prevent and manage missing incidents

A total of 19 of the 25 studies discussed technological devices; with most reporting that tracking devices help prevent or respond to missing episodes. Carers found these devices overall helpful [22, 26, 29, 40], noting reduced anxiety and enhanced independence for people with dementia. A key strength of technological solutions highlighted was the ability to track the person’s live location, providing reassurance and support:

‘*It is great that I had that comfort of knowing I could track my relative* [22]*.*’

‘*That helps reduce my anxiety about him being out by himself* [29].’

Healthcare professionals and device developers highlighted the role of technological devices in maintaining engagement in meaningful outdoor activities [23, 41, 42]. Such tools were perceived as not only supporting the health and well-being of the person with dementia but also as reducing carer burden:

‘*…the GPS device…enables somebody…being out and about, there’s the health benefits associated with that…the carer’s stress…considerably reduced* [23].’

Device developers observed that people with dementia emphasised the importance of early technology adoption to build familiarity and sustain engagement in independent activities as dementia progresses:

‘*I’ve met people that are wanting to start using…because they’re in early stage and they want to get used to something so that down the line, they’re ready* [41].’

Older people without dementia, healthcare professionals and technology representatives advocated for user-friendly designs that avoid stigmatisation [28, 43]. Key future research initiatives included improving ease of use, adapting devices to dementia stages [41, 44] and expanding usability testing [33].

Ethical considerations centred on consent and autonomy, with the need for people with dementia to choose device usage emphasised [25, 29]. People with dementia expressed discomfort with constant monitoring:

‘*I don’t want to be in a situation where big brother is watching me all the time…not comfortable with it* [25].’

They also expected that usual care should not be replaced by mobile phones or technology, and raised concerns about care being replaced by devices:

‘*I don’t want to feel as though my family are looking after me just through a mobile phone* [25].’

However, despite the strengths of using technological devices, several barriers were identified. These included limited knowledge around technologies by people with dementia and their carers [33], difficulties using digital platforms [22], lack of technological support [41] and concerns around device reliability and accuracy. Key technological limitations of tracking devices identified were a short battery life, the need to charge such devices constantly, as well as inconsistencies in location signals that could impact their reliability:

‘*Battery life (is short)…clients don’t always remember or know how to charge the devices, they require caregiver assistance* [22].’

‘*…due to signal blockage…lead to issues when trying to get the exact location…* [41].’

People with dementia expressed concern about device malfunctions that could place them at-risk, as carers might believe they are adequately protected:

‘*My device hasn’t been working for months…people around me weren’t checking on me because they thought I was protected, and I was not* [41].’

Technology was generally viewed as a tool but not a solution. Healthcare professionals and police officers emphasised that even the most advanced device could not fully replace human observation and community support:

‘*Although the device can locate older persons in real-time, it cannot assure that they are safe from other dangers, such as crossing the road* [45].’

## Discussion

### Summary of key findings

This review synthesised research on the lived experiences of people with dementia, their carers and other stakeholders in relation to safe walking and strategies to prevent missing episodes. People with dementia and their carers experienced emotional impacts following missing incidents, which may influence their attitudes towards engaging in outdoor walking. They acknowledged safe walking as essential for independence, well-being and quality of life, viewing it as supporting autonomy and preventing social isolation [42].

People with dementia relied on familiar wayfinding strategies [32] and adopted technological devices in early stages of dementia to facilitate future use [33, 42]. While live tracking provided carers with reassurance [22, 29], issues around privacy, consent, usability and ongoing support remain important to ethical and effective use [41]. They viewed these technologies as complementary to, rather than substitute for, dementia care, emphasising the importance of meaningful interpersonal engagement [25].

Healthcare professionals and police play key roles in supporting safe walking and preventing missing episodes among people with dementia. Their involvement includes follow-up visits after missing incidents [27, 39] and the use of specialised resources and search strategies to enhance search effectiveness [36], which carers perceived as beneficial [34]. However, carers often reported feelings of guilt, fear and concern over involving police [34], preferring neighbourly support, which was more available in rural than urban settings. In urban areas, community alert systems were described as beneficial when someone went missing [38], enabling immediate assistance despite limited neighbourhood connections.

### Interpretations of findings

This review highlights that people with dementia and their carers adopt diverse strategies—including wayfinding approaches, technological devices and engagement with police or community support—reflecting reliance on existing systemic measures. However, balancing autonomy in safe walking with carers’ concerns about potential risks remains a persistent challenge. Although technological devices can alleviate carers’ anxiety and assist in locating missing individuals, their adoption requires careful consideration of ethical consent, privacy and usability issues. An important contribution of our study therefore is highlighting that effective risk mitigation largely depends on personalised care plans complemented by technology that respect ongoing consent and autonomy [46]. Addressing these issues is crucial for the development of acceptable technology-based interventions [46, 47].

Carers’ help-seeking behaviour towards police was influenced by trust and emotional burden. In rural areas, stronger community ties supported neighbourly assistance, whereas such networks were often weaker in urban contexts. These findings highlight the need for multi-dimensional, context-sensitive interventions that incorporate carers’ preferences, community responses and the technology’s role in safeguarding people with dementia, by minimising stigma [16].

### Implication for practice

Our review highlights that psychoeducation and non-stigmatising support for carers are crucial to preventing harm associated with missing episodes [39]. Enhancing confidence in reporting incidents to police is equally important for effective risk management [9, 36]. As safe walking constitutes a vital component of post-diagnostic care and quality of life, dementia care packages should be grounded in a person-centred, rights-based approach [41].

Building on the lived experiences synthesised in this review, addressing the needs of people with dementia and their carers regarding missing incidents is essential for future care planning. As outdoor walking is highly valued, healthcare professionals should implement and regularly review personalised, safe walking plans across community and residential settings. Care plans should incorporate appropriate technologies and contingency measures to mitigate missing-related risks. Professionals should co-create safe walking strategies with people with dementia and their carers, ensuring these strategies are based on their views and preferences.

Integrating safe walking into dementia care is a complex process that necessities consideration of disease progression, daily living patterns and education for carers and professionals, while addressing ethical and technological constraints [39]. Effective risk mitigation strategies should be collaboratively developed, tailored to individual needs and risk profiles [48], reinforced by comprehensive assessments.

### Strengths and limitations of the synthesis

This review adopted a systematic, comprehensive search strategy across multiple databases to include high-quality qualitative studies. The use of narrative synthesis facilitated the integration of diverse qualitative data, thereby enhancing the conceptualisation of safe walking and risk mitigation strategies. Inclusion of studies from both community and residential care settings allowed for consideration of varying environmental contexts. However, excluding non-English studies may have introduced language bias. Additionally, the limited direct lived experience among certain participant groups—particularly people with dementia—resulted in insufficient and less diverse perspectives to fully capture the complexities of lived experiences [25, 28], affecting the applicability of the findings.

### Suggestions for future research and clinical practice

Safe walking is essential for people with dementia to maintain community engagement and preserve their quality of life. Future research would benefit from prioritising recruitment of people with dementia with direct experience of missing incidents or device use to enhance the validity of the qualitative synthesis and evaluate training tools for professionals undertaking follow-up visits after missing incidents and their impact on care practices. While technology may play a role in mitigating missing episodes, community support and access to care remain critical. Clinical trials reporting on personalised interventions to prevent harm from missing incidents in community and residential settings are urgently needed [49].

## Supplementary Material

Supplementary_materials_afaf371

## Data Availability

Data sharing does not apply to this article as no new data were generated or analysed in this narrative synthesis. All data supporting the findings of this study consist of previously published literature that is fully cited within the article’s reference list.
