# Supplementary material for: Supporting safe walking and managing missing incidents in dementia: a qualitative narrative synthesis of current evidence
Source: Age Ageing. 2025 Jan 3;55(1):afaf371. doi: 10.1093/ageing/afaf371 (PMC12763817; doi:10.1093/ageing/afaf371)
Supplement: Supplementary_materials_afaf371 [file supplementary_materials_afaf371.docx]

Supplementary Files

**Appendix 1. Search Strategy**

| # |  |
| --- | --- |
| S1 | (missing* or (missing* adj1 (incident or person* or case*)) or "go* missing*" or lost or "be* lost" or "get* lost" or wander* or "unattended wander*" or "wander* behavi*" or track* or "track* device*" or "search resource*" or "law enforcement" or elop* or "safe return*" or "police record*" or "search* and rescu*").ab,ti. |
| S2 | (dement* or Alzheimer* or "Alzheimer* disease" or "major cogniti* impair*" or "major cogniti* dis*" or "mild cogniti* impair*" or MCI).ab,ti. |
| S3 | S1 AND S2 |

**Appendix 2. Excluded studies (n = 40)**

|  | Reference | Reason for exclusion |
| --- | --- | --- |
| 1 | Niemeijer 2014 | Indoor walking but not missing |
| 2 | Aud 2000 | Not relevant to missing |
| 3 | Aud 2004 | Indoor walking but not missing |
| 4 | Hwang 1997 | Indoor walking but not missing |
| 5 | Alam 2023 | Indoor walking but not missing |
| 6 | Lester 2012 | Indoor walking but not missing |
| 7 | Giebel 2015 | Wrong methodology |
| 8 | Landau 2012 | Wrong methodology |
| 9 | McQuilkin 2016 | Indoor walking but not missing |
| 10 | Niemeijer 2015 | Indoor walking but not missing |
| 11 | McShane 1998 | Wrong methodology |
| 12 | Dugstad 2019 | Indoor walking but not missing |
| 13 | Dewing 2006 | Wrong methodology |
| 14 | Adekoya 2021 | Indoor walking but not missing |
| 15 | Dickinson 1998 | Indoor walking but not missing |
| 16 | Brorsson 2013 | Not relevant to missing |
| 17 | Wan 2016 | Wrong methodology |
| 18 | Adekoya 2019 | Indoor walking but not missing |
| 19 | Houston 2011 | Indoor walking but not missing |
| 20 | Thomas 1999 | Not relevant to missing |
| 21 | Chen 2012 | Wrong outcome |
| 22 | Bantry-White 2016 | Wrong methodology |
| 23 | Thomas 1997 | Wrong methodology |
| 24 | Ward 2022 | Wrong outcome |
| 25 | MacAndrew 2019 | Wrong setting |
| 26 | Neubauer 2019a | Poster abstract |
| 27 | RibasMiquel 2015 | Supplementary |
| 28 | Hovland 2021 | Not relevant to missing |
| 29 | MacAndrew 2017 | Indoor walking but not missing |
| 30 | Eikelboom 2020 | Wrong outcome |
| 31 | Thomas 1996 | Wrong methodology |
| 32 | Elprama 2016 | Indoor walking but not missing |
| 33 | Bonin-Guillaume 2017 | Wrong outcome |
| 34 | O'Donnell 2023 | Wrong methodology |
| 35 | Landau 2009 | Wrong methodology |
| 36 | Neubauer 2019b | Poster abstract |
| 37 | Hope 1990 | Indoor walking but not missing |
| 38 | Brittain 2017 | Wrong outcome |
| 39 | Bantry-White 2010 | Duplicate sample (earlier publication) |
| 40 | Ah Yoo 2025 | Duplication |

**Appendix 3. CASP Qualitative Checklist Scores**

| Study | CASP01 | CASP02 | CASP03 | CASP04 | CASP05 | CASP06 | CASP07 | CASP08 | CASP09 | CASP10 | Total Score |
| --- | --- | --- | --- | --- | --- | --- | --- | --- | --- | --- | --- |
| Shalev-Greene *et al.* [34] | 1 | 1 | 1 | 1 | 1 | 1 | 1 | 1 | 1 | 1 | **10** |
| Kearns *et al.* [28] | 1 | 1 | 1 | 1 | 1 | 0 | 0 | 1 | 1 | 1 | **8** |
| Landau *et al.* [45] | 1 | 1 | 1 | 1 | 1 | 0 | 0 | 1 | 1 | 1 | **8** |
| Neubauer *et al.* [16] | 1 | 1 | 1 | 1 | 1 | 0 | 1 | 1 | 1 | 1 | **9** |
| Neubauer and Liu. [24] | 1 | 1 | 1 | 1 | 1 | 0 | 1 | 1 | 1 | 1 | **9** |
| *Bantry-White *et al.* [29] | 1 | 1 | 1 | 1 | 1 | 0 | 1 | 1 | 1 | 1 | **9** |
| Robinson *et al.* [25] | 1 | 1 | 1 | 1 | 1 | 0 | 1 | 1 | 1 | 1 | **9** |
| Robinson *et al.* [30] | 1 | 1 | 1 | 1 | 1 | 0 | 1 | 1 | 1 | 1 | **9** |
| Dickson*.* [31] | 1 | 1 | 1 | 1 | 1 | 1 | 0 | 1 | 1 | 1 | **9** |
| Mahoney and Mahoney*.* [43] | 1 | 1 | 1 | 1 | 1 | 0 | 0 | 0 | 1 | 1 | **7** |
| Milne *et al.* [23] | 1 | 1 | 1 | 1 | 1 | 0 | 0 | 0 | 1 | 1 | **7** |
| Howes *et al.* [42] | 1 | 1 | 1 | 1 | 1 | 1 | 1 | 1 | 1 | 1 | **10** |
| Dodds. [37] | 1 | 1 | 1 | 1 | 1 | 0 | 1 | 0 | 1 | 1 | **8** |
| Rasquin *et al.* [33] | 1 | 1 | 1 | 1 | 1 | 0 | 0 | 0 | 1 | 1 | **7** |
| Neubauer *et al.* [41] | 1 | 1 | 1 | 1 | 1 | 0 | 0 | 1 | 1 | 1 | **8** |
| Neubauer *et al.* [44] | 1 | 1 | 1 | 1 | 1 | 0 | 1 | 1 | 1 | 1 | **9** |
| Liu *et al.* [22] | 1 | 1 | 0 | 1 | 1 | 0 | 0 | 0 | 1 | 1 | **6** |
| Olsson *et al.* [32] | 1 | 1 | 1 | 1 | 1 | 0 | 1 | 1 | 1 | 1 | **9** |
| Adekoya *et al.* [38] | 1 | 1 | 1 | 1 | 1 | 0 | 1 | 1 | 1 | 1 | **9** |
| Ah Yoo *et al.* [36] | 1 | 1 | 1 | 1 | 1 | 0 | 0 | 1 | 1 | 1 | **8** |
| Doyle *et al.* [26] | 1 | 1 | 1 | 1 | 1 | 0 | 1 | 1 | 1 | 1 | **9** |
| Hu *et al.* [39] | 1 | 1 | 1 | 1 | 1 | 0 | 1 | 1 | 1 | 1 | **9** |
| Letts *et al.* [27] | 1 | 1 | 1 | 1 | 1 | 0 | 1 | 1 | 1 | 1 | **9** |
| Li *et al.* [35] | 1 | 1 | 1 | 1 | 1 | 0 | 1 | 1 | 1 | 1 | **9** |
| Löbe and Petersen. [40] | 1 | 1 | 1 | 1 | 1 | 0 | 1 | 1 | 1 | 1 | **9** |

Yes = 1 No/Can’t Tell = 0

CASP includes the following qualitative checklist questions: 1. Was there a clear statement of the aims of the research? 2. Is a qualitative methodology appropriate? 3. Was the research design appropriate to address the aims of the research? 4. Was the recruitment strategy appropriate to the aims of the research? 5. Were the data collected in a way that addressed the research issue? 6. Has the relationship between researcher and participants been adequately considered? 7. Have ethical issues been taken into consideration? 8. Were the data analyses sufficiently rigorous? 9. Is there a clear statement of findings? 10. How valuable is the research?

*This study used the same sample as Bantry-White *et al.* (2010); the earlier publication was excluded to avoid duplication.
